# Effective treatment for massive neonatal catheter-related right atrial thrombosis

**DOI:** 10.1093/icvts/ivac055

**Published:** 2022-03-09

**Authors:** Shuji Gong, Yifeng Yang, Mingliang Tan, Jinlan Chen

**Affiliations:** Department of Cardiovascular Surgery, The Second Xiangya Hospital of Central South University, Changsha, China

**Keywords:** Catheter-related right atrial thrombosis, Congenital heart disease, Surgical thrombectomy

## Abstract

Catheter-related thrombosis is a common complication caused by central venous catheters. Although right atrial thrombosis is uncommon, it may lead to life-threatening situations. Here, we report 2 cases of neonates with massive catheter-related right atrial thrombosis after congenital heart disease surgery. During therapeutic management, we attempted different treatments but failed to clear the mass. Finally, we found thrombectomy to be the most effective method for treating massive catheter-related right atrial thrombosis with favourable results.

## INTRODUCTION

For critically ill newborns, central venous catheters (CVCs) are crucial for receiving nutrition and medicine. However, the use of CVCs may cause severe complications, such as infection, thrombosis and technical complications. Catheter-related right atrial thrombosis (CRAT) is uncommon but can lead to life-threatening situations such as the superior vena cava syndrome and pulmonary embolism [1]. In the present study, we share our successful surgical experience in treating CRAT in neonates.

## CASE REPORT

### Case 1

**Figure 1:**
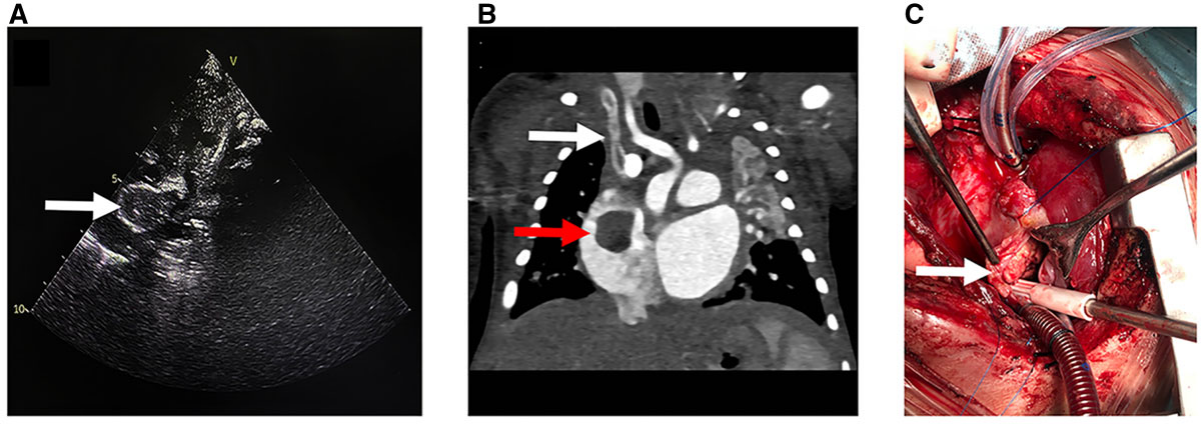
Imaging and surgery pictures. (**A**) Transthoracic echocardiogram demonstrating a large right atrial mass (arrow). (**B**) Right atrial mass (red arrow) and central venous catheters (white arrow) as seen on computed tomography (CT). (**C**) A right atrial thrombus was seen during surgery (arrow).

A male neonate (weight 3.55 kg, length 51 cm) with congenital heart disease underwent corrective cardiac surgery (ventricular septal defect repair, atrial septal defect repair, ligation of the patent ductus arteriosus and interrupted aortic arch repair) on the 5th day after birth. After the operation, the newborn was transferred to the paediatric cardiac intensive care unit with a CVC placed through the right jugular vein to the right atrium. Dopamine and adrenaline were administered via the CVC, and heparin was not used prophylactically. The CVC was not removed until a giant thrombus in the right atrium was detected after routine echocardiography on the 28th postoperative day. The thrombus was ∼30 mm × 15 mm in size and occupied over half the entire right atrium (Fig. 1A). Computed tomography angiography was performed to confirm the thrombus formation (Fig. 1B). Blood culture test results were positive for *Enterococcus faecalis*. Antibiotic therapy based on susceptibility results was provided. Thrombolytic therapy was administered with recombinant tissue plasminogen activator (0.5 mg/kg/h for 6 h) followed by anticoagulant therapy. Heparin was administered continuously via a peripheral venous line for 3 days to maintain the activated partial thromboplastin time at 50–55 s, combined with 2 mg/kg of aspirin tablet once a day. Thrombolytic therapy was administered via recombinant tissue plasminogen activator, followed by anticoagulant therapy with heparin and aspirin. However, the therapeutic results were unsuccessful. Thus, surgical thrombectomy was planned and successfully performed (Fig. 1C) on the 45th postoperative day. The patient was discharged on the 60th postoperative day with no thrombus formation during the 1-year follow-up period.

### Case 2

A 1-month-old male neonate (birth weight 2.13 kg, length 47 cm) was admitted to the paediatric cardiac intensive care unit after corrective surgery for congenital heart disease (ventricular septal defect repair, atrial septal defect repair and correction of aortic coarctation). A CVC was inserted through the right jugular vein on the 10th postoperative day for nutrition and medicine. No heparin was used as in case 1. A thrombus in the right atrial chamber was diagnosed when echocardiography was performed as a part of the routine follow-up on the 25th postoperative day. The thrombus was massive (20 mm × 15 mm) and almost blocked the superior vena cava inlet to the right atrium, but the neonate was asymptomatic due to a persistent left superior vena cava. Thrombectomy was not performed after diagnosis because the neonate was notably weak and could not tolerate a second operation. Thrombolytic and anticoagulant therapies were administered for 12 h and 7 days, respectively, and their plans were the same as in case 1. Thrombolytic therapy followed by anticoagulant therapy as case 1 was administered. Echocardiography was performed weekly. The thrombus reduced in size but still obstructed the right superior vena cava. One month later, when the baby’s condition improved, the thrombus was surgically removed. We could not completely remove the thrombus in the right superior vena cava because it was extensive, so we chose to ligate the right superior vena cava during the operation. The child had mild oedema early after surgery, but the symptoms subsequently disappeared. Computed tomography angiography also revealed a wide left superior vena cava (Supplementary Material 1). The development of the baby was delayed, but no thrombosis was present at the 10-month follow-up.

## DISCUSSION

Although clinical cases concerning the management and treatment of CRAT in adults have been reported in the literature, CRATs in newborns are seldom mentioned and are rarely reported in China. To remove intracardiac thrombi effectively and safely once CRAT is diagnosed, medical management should be considered as the first treatment; however, there is no prospective experimental evidence to support this notion [2]. For large or infected CARTs, surgery should actively be considered, especially after the failure of anticoagulation and thrombolysis. Lalor and Sutter suggested open thrombectomy as an optimal and definitive treatment in large (>2 cm) mobile CRATs with adherence to the catheter and atrial wall, in patients who show signs of infection or superior vena cava obstruction and in low-risk surgical candidates [3]. van Laecke *et al.* [4] opined that surgery should be performed when the thrombus is larger than 2 cm or if there is evidence of infection. In our case, the thrombi were extensive and adhered to the central line. In case 2 particularly, the superior vena cava was occluded by the thrombus. Anticoagulation and thrombolysis therapies were attempted but failed to resolve the thrombi. Surgical removal of the right atrial thrombus was successfully performed in both cases.

To prevent CARTs in newborns after surgery, risk factors for catheter-related thrombus, including catheter position, catheter type and abnormal coagulation function should first be avoided [5]. Second, regular reviews and checks need to be conducted in a timely fashion after inserting the CVCs. Third, catheters should be removed as early as possible as they are risk factors for thrombosis. Some experts recommend anticoagulation prophylaxis after CVC placement, while others hold the opposite view [5]. According to the experience at our centre, if the CVC is only used for a short period and there is continuous fluid input, anticoagulation is not required.

In conclusion, early removal of catheters should be the first step in the management of CVCs. Once CRATs are identified, CVCs should be removed as soon as possible. However, it is unclear which management policy is the best for massive CART, but based on this report and our experience, surgical thrombectomy is highly effective for large right atrial thrombi.

## SUPPLEMENTARY MATERIAL

Supplementary material is available at *ICVTS* online.

**Conflict of interest:** none declared.

### Reviewer information

Interactive CardioVascular and Thoracic Surgery thanks Gunter Kerst and the other, anonymous reviewer(s) for their contribution to the peer review process of this article.

## Supplementary Material

ivac055_Supplementary_Data
