# Supplementary figures and images for: Effective treatment for massive neonatal catheter-related right atrial thrombosis
Source: Interact Cardiovasc Thorac Surg. 2022 Mar 9;35(1):ivac055. doi: 10.1093/icvts/ivac055 (PMC9714592; doi:10.1093/icvts/ivac055)

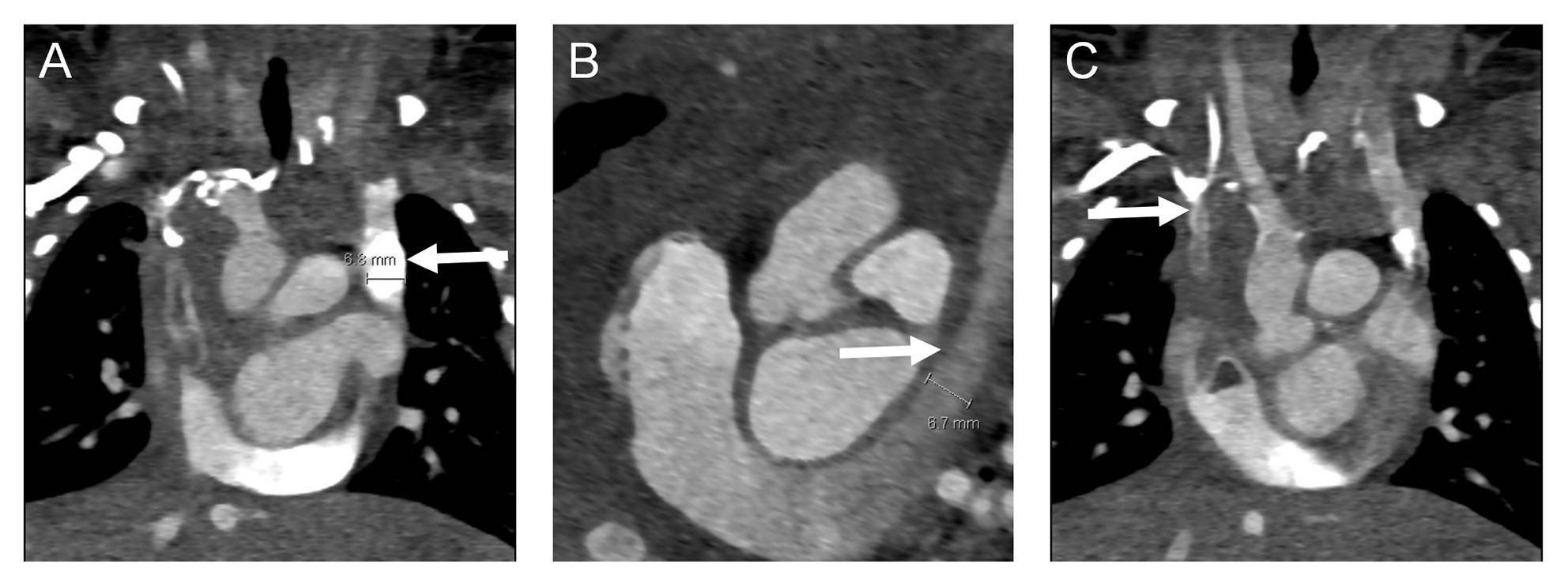

Supplement: ivac055_Supplementary_Data [file ivac055_supplementary_data.jpeg]
